# Tetra-block: ultrasound femoral, lateral femoral-cutaneous, obturator, and sciatic nerve blocks in lower limb anesthesia: a case series

**DOI:** 10.1186/s13256-023-04017-6

**Published:** 2023-07-01

**Authors:** Antonio Coviello, Carmine Iacovazzo, Dario Cirillo, Pasquale Diglio, Alessio Bernasconi, Andrea Cozzolino, Antonio Izzo, Annachiara Marra, Giuseppe Servillo, Maria Vargas

**Affiliations:** 1 Department of Neurosciences, Reproductive and Odontostomatological Sciences, University of Naples “Federico II”, Via Sergio Pansini, 5, Napoli NA, 80131 Naples, Italy; 2 Unit of Orthopedics and Traumatology, Department of Public Health, School of Medicine, University of Naples “Federico II”, Naples, Italy

**Keywords:** Case series, Locoregional anesthesia, Femoral nerve block, Lateral femoral cutaneous nerve block, Obturator nerve block, Sciatic nerve block

## Abstract

**Background:**

The gold standard anesthesiologic procedure for urgent femur fracture surgery is Spinal Anesthesia. It is not always feasible because of patients' severe comorbidities and difficulties in optimizing drug therapy in the appropriate time frame such as discontinuation of anticoagulant drugs. The use of four peripheral nerve blocks (tetra-block) can be a winning weapon when all seems lost.

**Case presentation:**

We present, in this case series, three Caucasian adult femur fractures (an 83-year-old woman, a 73-year-old man, and a 68-year-old woman) with different and major comorbidities (cardiac or circulatory disorders on anticoagulants therapy that were not discontinued on time; breast cancer and others) underwent the same anesthesiologic approach in the urgent setting. Ultrasound peripheral nerve blocks, that is femoral, lateral femoral cutaneous, obturator, and sciatic with parasacral approach were successfully performed in all patients who underwent intramedullary nailing for intertrochanteric fracture. We evaluated the adequacy of the anesthesia plane, postoperative pain control with the VAS scale, and the incidence of postoperative side effects.

**Conclusions:**

Four peripheral nerve blocks (Tetra-block) can be alternative anesthesiologic management in urgent settings, in patients where drug therapy cannot be optimized, as in antiplatelet and anticoagulant therapy.

## Background

Femur fracture (FF) represents an event with disabling outcomes in elderly patients, with a significant impact on the quality of life and public health in general.

FF in the elderly is, in most cases, the result of an accidental fall or even minor trauma and is often associated with osteoporosis/low bone mass and other conditions [1] such as functional impairment of the lower limbs, Parkinson's disease, and visual impairment, [2] which can considerably increase the risk of falls. A Systematic Review of 72 studies carried out in 63 different countries revealed that Italy is among the countries with the highest incidence of hip fractures, annually recording an incidence of > 300 per 100,000 inhabitants for women and > 150 for men [3].

In addition to increased mortality in elderly patients, [4] FF sometimes has a devastating impact on quality of life, leading to the risk of reduced mobility with limitation or loss of autonomy and the inability to return to pre-trauma conditions [5]. Guidelines of Italian Society for Orthopedics and Traumatology (SIOT) recommend surgery for patients with FF on the day of arrival at the hospital (within 24 h of arrival) or, at the latest, the next day (within 48 h of arrival) [6].

The anesthetic approach to orthopedic surgery can be General Anesthesia (GA), Neuraxial Anesthesia (NA), and Loco-Regional Anesthesia (LRA). The literature does not definitively clarify the statistically significant difference between GA and Peripheral Nerve Blocks (PNB) for mortality and postoperative complications [7]. Nevertheless, some studies clearly show lower mortality after 30 days in patients undergoing Regional Anesthesia (RA) with PNB and highlight better pain control in the postoperative period, better hemodynamic stability, faster functional recovery, and a clear reduction in respiratory complications associated with GA [8].

Ultrasound femoral, lateral femoral-cutaneous, obturator, and sciatic (parasacral approach) nerve blocks (tetra-block) can be an efficient anesthesiologic approach when all seems lost in patients undergoing surgical femur fracture treatment. This anesthesiologic management showed several advantages including reduced hemodynamic impact because this technique had only affected the area of the body that undergoing the surgery, reduced the risk of post-operative fatigue and vomiting as well as improved post-operative pain management. Ultrasound peripherical nerve blocks are a safe approach in the hands of clinicians with considerable experience in this area. The proposed anesthetic approach may be particularly suitable for fragile patients or for those who could obtain greater benefits, given their comorbidities, from opioid-free anesthesia [9]. NA is not always feasible, both due to the lack of pharmacological optimization in patients who are often subject to anticoagulant therapy and the difficulty to position the patient himself, as well as the pain felt. The approach with LRA can be considered valid provided that it is performed by expert operators since the Local Anesthetic Systemic Toxicity (LAST) could be the greatest risk to encounter [10].

We propose the cases of three patients with multiple comorbidities, candidates for urgent surgery, for whom pharmacological optimization could not occur.

## Case presentation

The patients agreed to the use of their data in the publication of this case series for scientific and clinical purposes. Ethics committee approval was not sought for this retrospective study since we analyzed data collected during routine clinical practice.

### Case 1

**Fig. 1:**
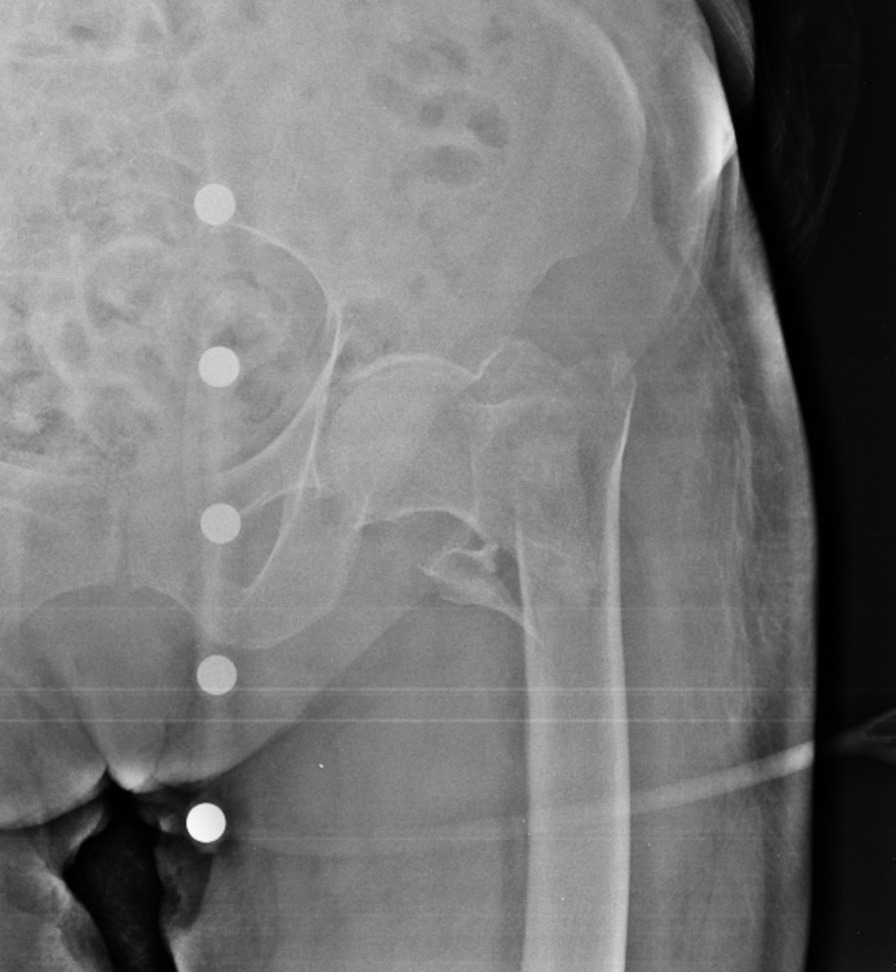
Preoperative anteroposterior radiograph of the left hip demonstrating displaced intertrochanteric femur fracture

**Fig. 2:**
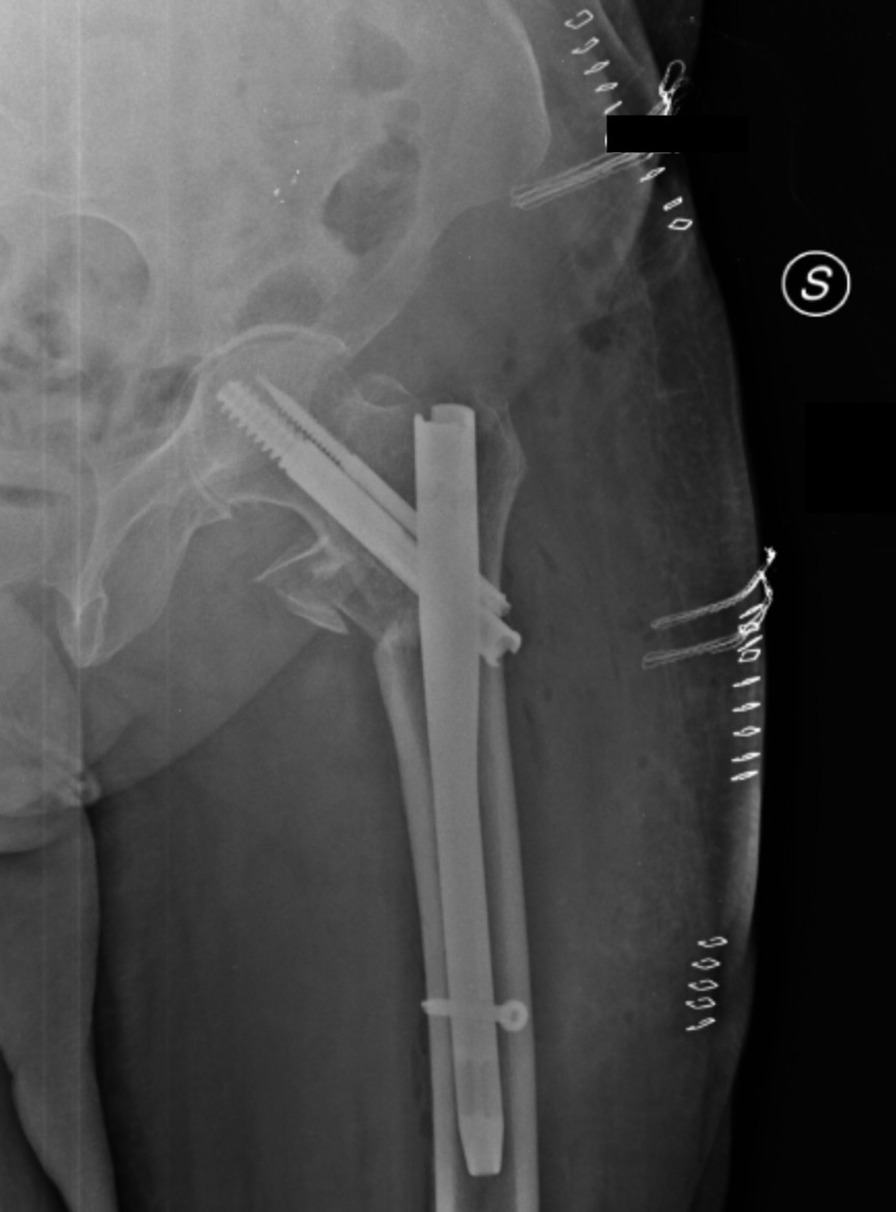
Postoperative anteroposterior radiograph of the left hip

An 83-year-old Caucasian woman (Body Mass Index (BMI), 27.34 kg/m^2^; weight, 70 kg; height, 160 cm) suffered from Type 2 Diabetes Mellitus, Chronic Obstructive Pulmonary Disease (COPD) with pleurogenic results, left pulmonary lobectomy, Chronic Kidney Disease III stage, persistent Atrial Fibrillation under treatment, Ejection Fraction (EF) 30% and a previous right femur fracture arrived to the emergency room of a small town following a fall during the night. The patient presented evident deformity of the left lower limb, so an x-ray was performed which showed AO/OTA 31A2 type left intertrochanteric fracture (Fig. 1) [11, 12]. In order to contain transport times, the patient was transported by helicopter to the base of the Cardarelli Hospital of Naples (Italy) and subsequently admitted to the Orthopedics and Traumatology Department of Federico II University Hospital of Naples (Italy). The patient's pharmacotherapy also included Novel Oral Anticoagulants (NAO), normally taken by the patient a few hours before the fall. The preoperative hemoglobin level was 9.0 g/dl. Given the orthopedic surgical treatment of intramedullary nailing of the left femur (Fig. 2) that could cause major blood loss, Erythropoietin (450 UI/kg), and Ferinject (20 mg/kg), dosed according to the patient’s weight, were administered. PNB, the anesthetic technique choice, was performed with Mepivacaine 1% 200 mg, and Ropivacaine 0.375% 75 mg plus Dexamethasone 8 mg.

### Case 2

A 73-year-old Caucasian man (BMI, 30.64 kg/m2; weight, 96 kg; height, 177 cm), who suffered from Type 2 Diabetes Mellitus, Hypertension, severe Aortic Stenosis, and previous Covid19 infection, arrived at the emergency room of a local hospital due to chest and right hip pain after accidental fall caused by syncopal episode. After practicing the appropriate diagnostic tests NSTEMI was diagnosed.

**Fig. 3:**
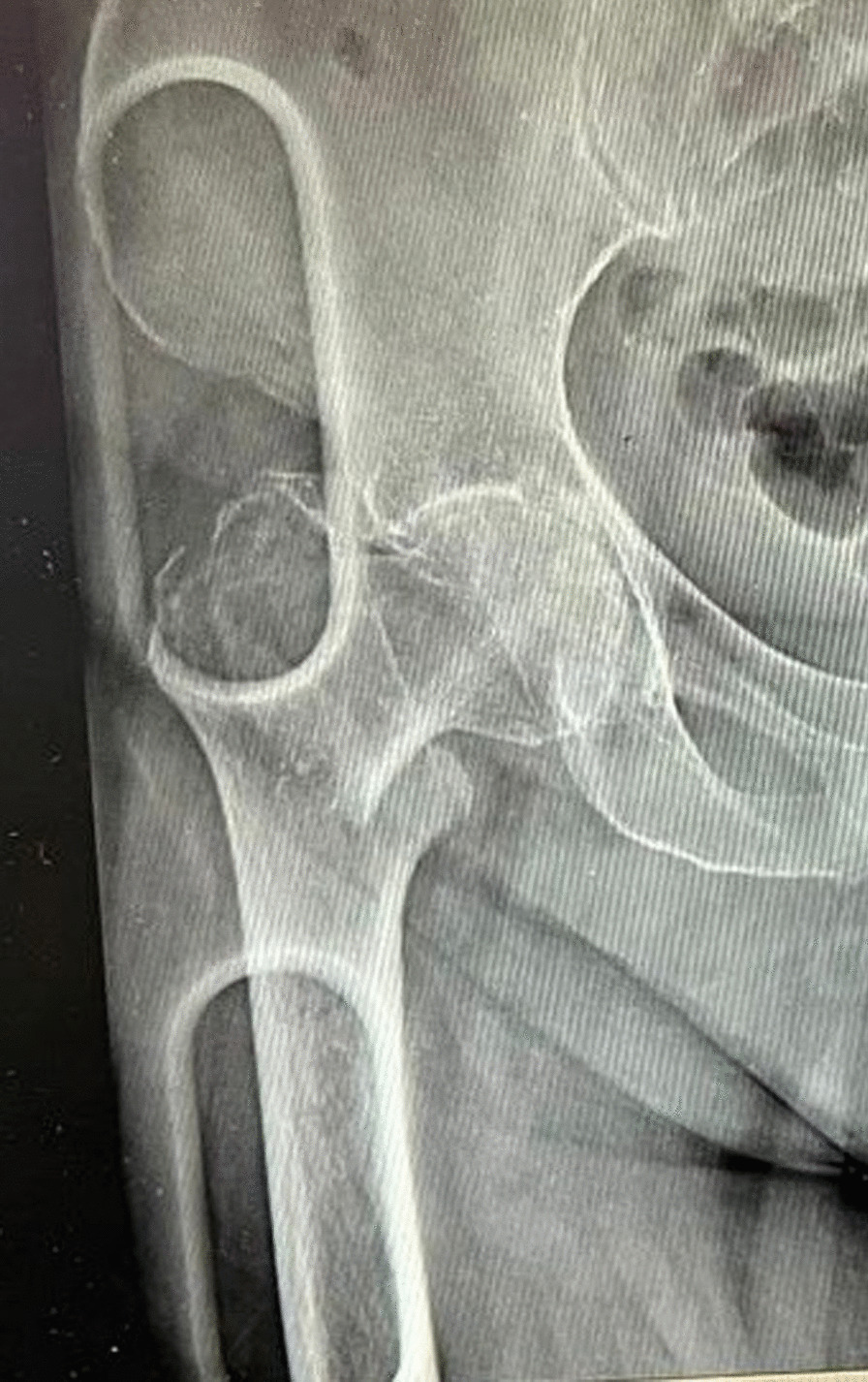
Preoperative anteroposterior radiograph of the right hip demonstrating displaced intertrochanteric femur fracture

**Fig. 4:**
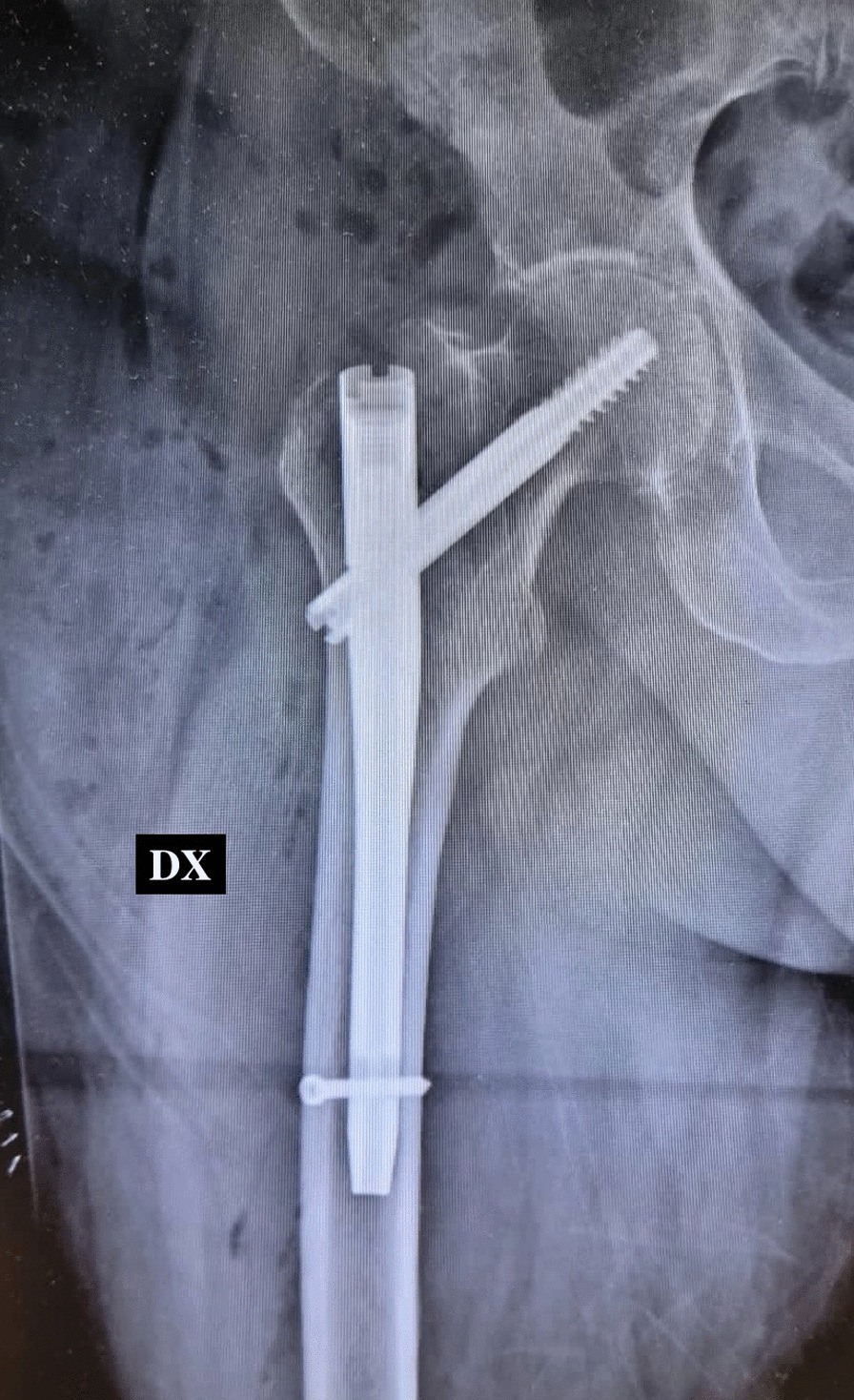
Postoperative anteroposterior radiograph of the right hip

In addition, the x-ray of the painful hip showed AO/OTA 31A1.2 type right intertrochanteric fracture (Fig. 3) [11, 12]. The patient was immediately transferred to the Cardiology, Hemodynamics, and Cardiac Intensive Care Unit (CICU) of Federico II University Hospital of Naples (Italy) where he underwent a revascularization procedure with the use of drug-eluting stents, heart failure after myocardial infarction (EF < 21%) The patient took pharmacotherapy for his pathologies and was on Dual Antiplatelet Therapy (DAPT) for the acute cardiac event. After a multidisciplinary meeting between the anesthesiologist, cardiologist, and orthopedic surgeon, it was decided to suspend Ticagrelor 4 days earlier and switch it with Tirofiban, which had to be discontinued 4 h before surgery. The preoperative hemoglobin level was 8.2 g/dl. Given the orthopedic surgical treatment of intramedullary nailing of the right femur that could cause major blood loss, Ferinject (20 mg/kg) dosed—considering the patient’s weight—was administered. Next, he underwent orthopedic surgical treatment of intramedullary nailing of the right femur (Fig. 4). PNB, the anesthetic technique choice, was performed with Mepivacaine 1% 200 mg, and Ropivacaine 0.375% 75 mg plus Dexamethasone 8 mg.

### Case 3

**Fig. 5:**
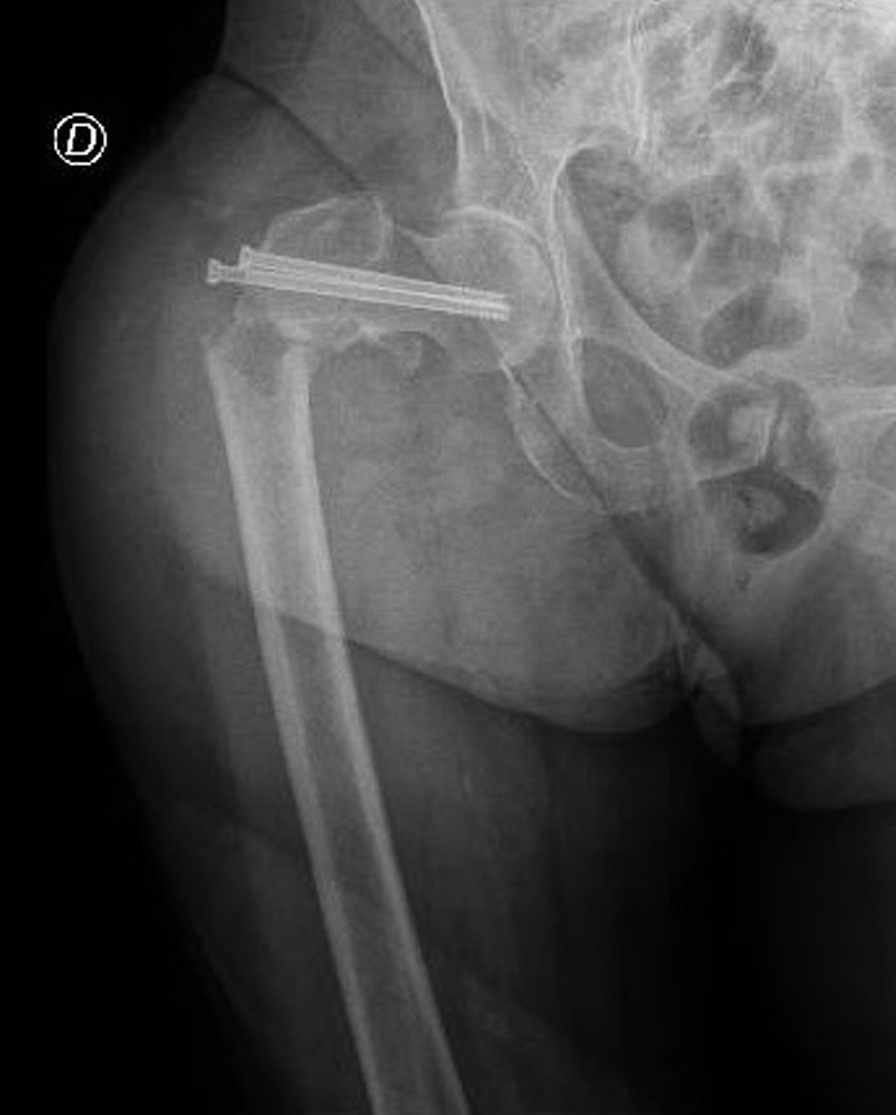
Preoperative anteroposterior radiograph of the right hip demonstrating displaced intertrochanteric femur fracture

**Fig. 6:**
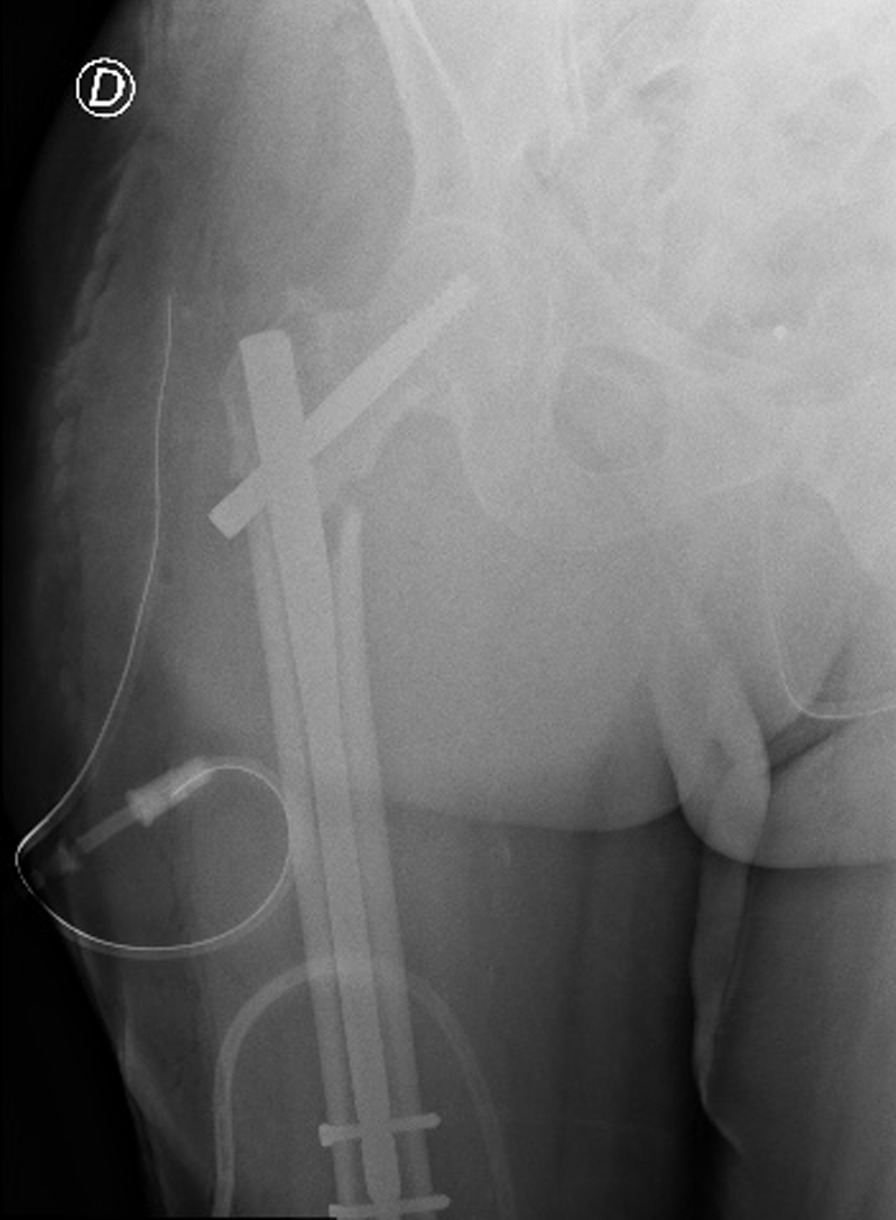
Postoperative anteroposterior radiograph of the right hip

A 68-year-old Caucasian woman (BMI, 37.29 kg/m2; weight, 104 kg; height, 167 cm) suffered from Hypertension, Toxic Multinodular Goiter, Dyslipidemia, Obesity class II, Chronic Kidney Disease III stage and Invasive Ductal Carcinoma (IDC) with the convolution of metastasis to the vertebral column by D9 to L5, lungs, and bones. The patient presented evident deformity of the right lower limb, so an x-ray was performed. It showed AO/OTA 31A3.1 type right intertrochanteric fracture (Fig. 5) [11, 12]. It was a pathological fracture; It was a pathological fracture, that is a spontaneous fracture not secondary to a traumatic event while she was admitted to our Oncology Department of Federico II University Hospital of Naples (Italy) for the treatment of the carcinoma, subsequently admitted to the Orthopedics and Traumatology Department to undergo surgical treatment of intramedullary nailing of the right femur (Fig. 6). In addition, the patient had extensive deep vein thrombosis of the medial twin vein and the left popliteal vein. The patient's pharmacotherapy also included Fondaparinux 10 mg subcutaneously once daily. PNB, the anesthetic technique choice, was performed with Mepivacaine 1% 200 mg, and Ropivacaine 0.375% 75 mg plus Dexamethasone 8 mg.

### Anesthesiologic management

In the operating room, venous access was placed (16 or 18 Gauge) and antibiotic prophylaxis was administered (Cefazolin 1 or 2 gr. iv, or in case of allergy, Clindamycin 600 mg iv). Pantoprazole 40 mg iv was also administered. Pulse oximetry (SpO2), heart rate (HR), body temperature (C°), continuous invasive arterial (cIBP), and cerebral oximetry with ForeSight were monitored. Pre-load was performed with crystalloid 500 mL and Ondansetron 8 mg, and pre-procedural sedation was performed with Midazolam 0.01–0.03 mg/Kg. The patients underwent PNB and intra-operative sedation with Dexmedetomidine 0.7 gamma/Kg/h. All patients received O2-therapy with a nasal cannula with a flow rate of 2L/min.

### Ultrasound peripherical nerve block

#### *Ultrasound femoral nerve block (*Fig. 7*)*

**Fig. 7:**
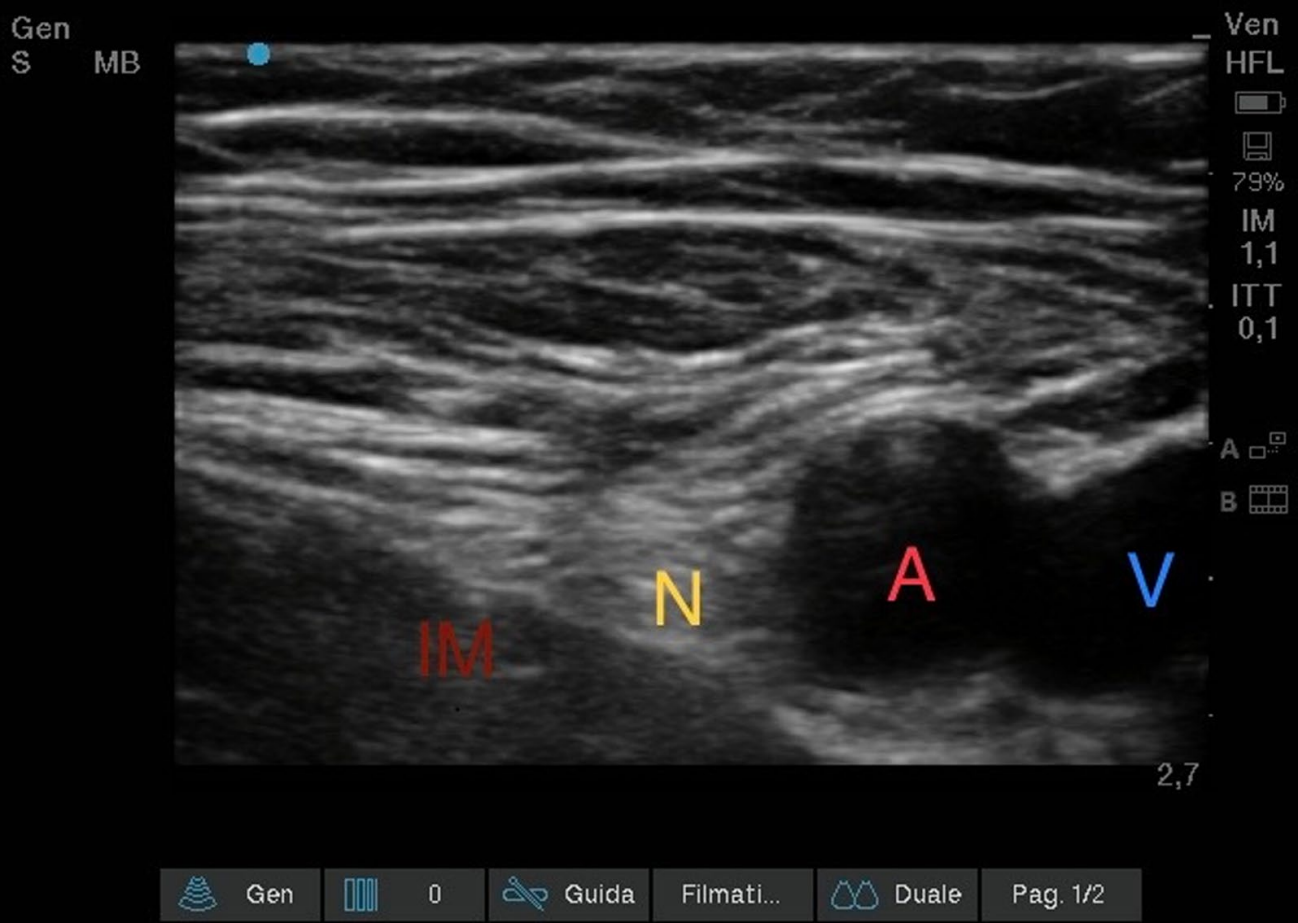
Ultrasound Femoral nerve block. Iliac muscle (IM); Femoral nerve (N); Femoral artery (A); Femoral vein (V)

The patient was positioned supine with the legs slightly abducted: the inguinal and thigh region were disinfected and sterile towels were placed to delimit the field. The ultrasound device was positioned contralaterally to the limb to be treated. The physician applied the probe to the patient's groin with the point of the probe conventionally placed on the right side of the patient. Using an ultrasound (US) transducer (Sonosite HLF38 × 13–6 MHz, Fujifilm Sonosite Europe, Amsterdam, Netherlands), the inguinal ligament was visualized as a hyperechoic structure; sliding in the caudal direction with the probe, the femoral vein, compressible, and the femoral artery, non-compressible and pulsatile, were identified. It was scrolled laterally until the femoral nerve was identified, which appeared like a hyperechoic triangle. From the lateral side of the transducer (lateral-to-medial orientation), an 85-mm long 21-Gauge 30° tip (Vygon Value Life, Italy) to ensure complete visualization of the needle tip, was inserted. The correct position of the needle tip was double-checked through the progressive injection of 3 ml of Saline Solution. An anesthetic solution of Ropivacaine 0.375% (dose 26.25 mg) plus Mepivacaine 1% (dose 70 mg) and Dexamethasone 4 mg, total volume 15 ml, was subsequently injected until a complete detachment of the iliac fascia.

#### Ultrasound lateral femoral-cutaneous nerve block (Fig. 8)

**Fig. 8:**
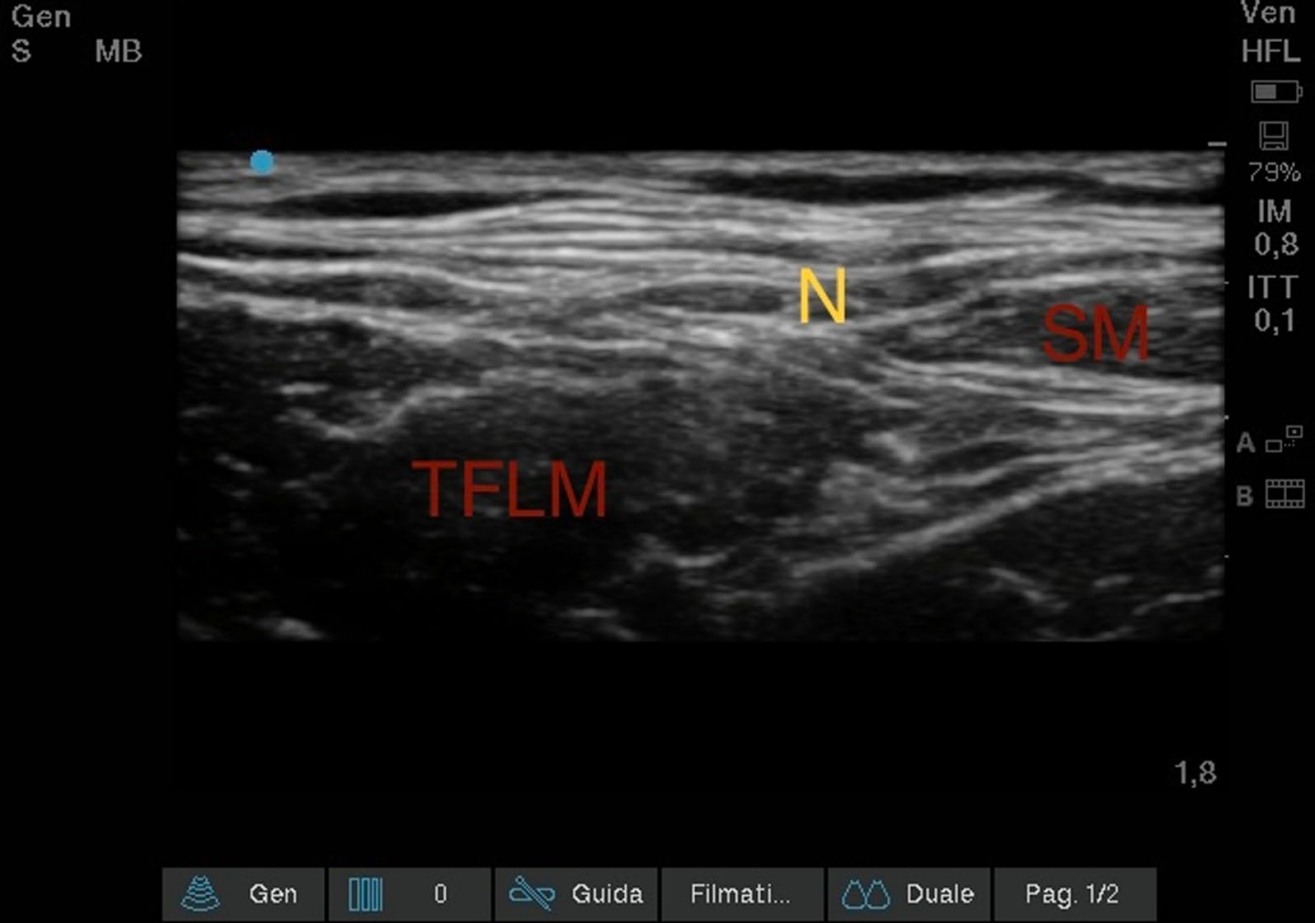
Ultrasound Lateral Femoral-Cutaneous Nerve Block. Sartorius muscle (SM); Tensor fasciae latae muscle (TFLM); Lateral Femoral-Cutaneous nerve (N)

Once the Femoral Nerve block was completed, we proceeded with a lateral scrolling of the probe and we went to search for the compartment that was created between the Fascia Lata, the Tensor Muscle of the Fascia Lata and the Sartorio Muscle. There we could visualize the Lateral Femoral-Cutaneous Nerve with the characteristic "eye" appearance. From the lateral side of the transducer (lateral-to-medial orientation), an 85-mm long 21-Gauge 30° tip was inserted (Vygon Value Life, Italy) to ensure complete visualization of the needle tip. An anesthetic solution of Ropivacaine 0.375% (dose 1.875 mg) plus Mepivacaine 1% (dose 5 mg), total volume 1 ml, was subsequently injected.

#### *Ultrasound obturator nerve block (*Fig. 9*)*

**Fig. 9:**
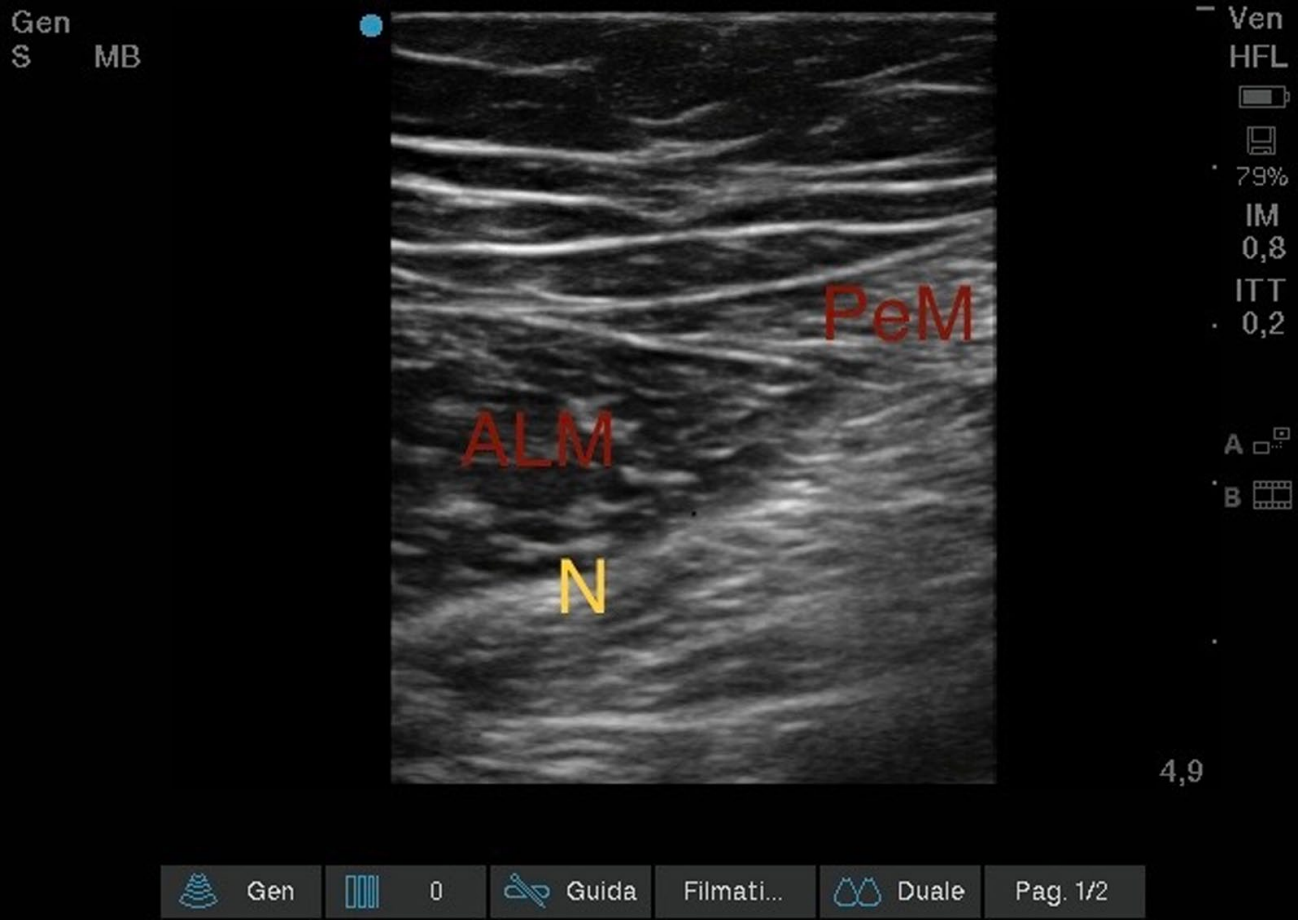
Ultrasound obturator nerve block. Adductor longus muscle (ALM); Pectineus muscle (PeM); Anterior branch of obturator nerve (N)

Starting again from the patient's groin, after visualizing the femoral artery and vein, the probe was scrolled 2–4 cm caudally and later medially, until the triple layering of the Adductor Longus, Adductor Brevis and Adductor Grande Muscles were visualized. The visualization of the hyperechoic intermuscular septa allowed the branches of the obturator nerve to be shown as flat, oval structures. From the lateral side of the transducer (lateral-to-medial orientation), an 85-mm long 21-Gauge 30° tip (Vygon Value Life, Italy) was inserted to ensure complete visualization of the needle tip. The correct position of the needle tip was double-checked through the progressive injection of 3 ml of Saline Solution. An anesthetic solution of Ropivacaine 0.375% (dose 9.375 mg) plus Mepivacaine 1% (dose 25 mg), total volume 5 ml, was later injected. (Fig. 6).

#### *Ultrasound sciatic nerve block (*Fig. 10–11*)*

**Fig. 10:**
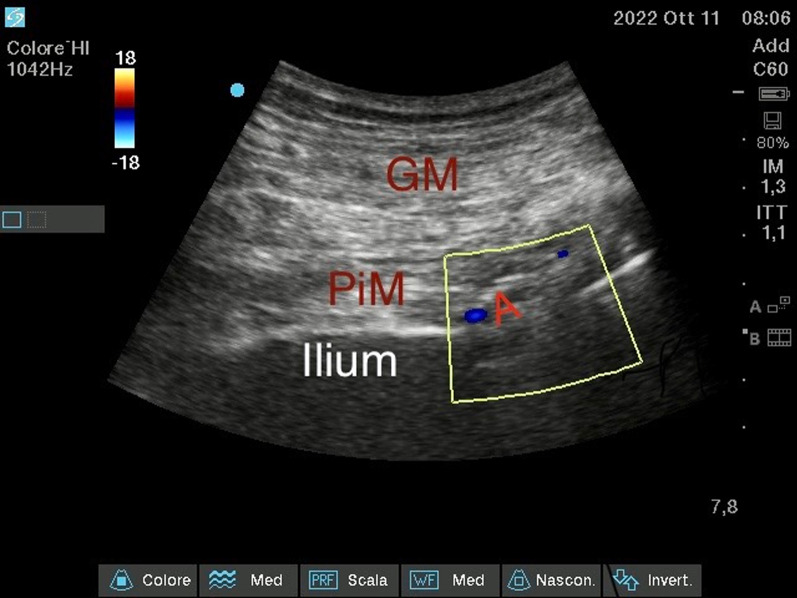
Ultrasound Sciatic Nerve block—Parasacral approach—ecocolordoppler image. Gluteal muscle (GM); Piriformis muscle (PiM); Gluteal artery (A)

**Fig. 11:**
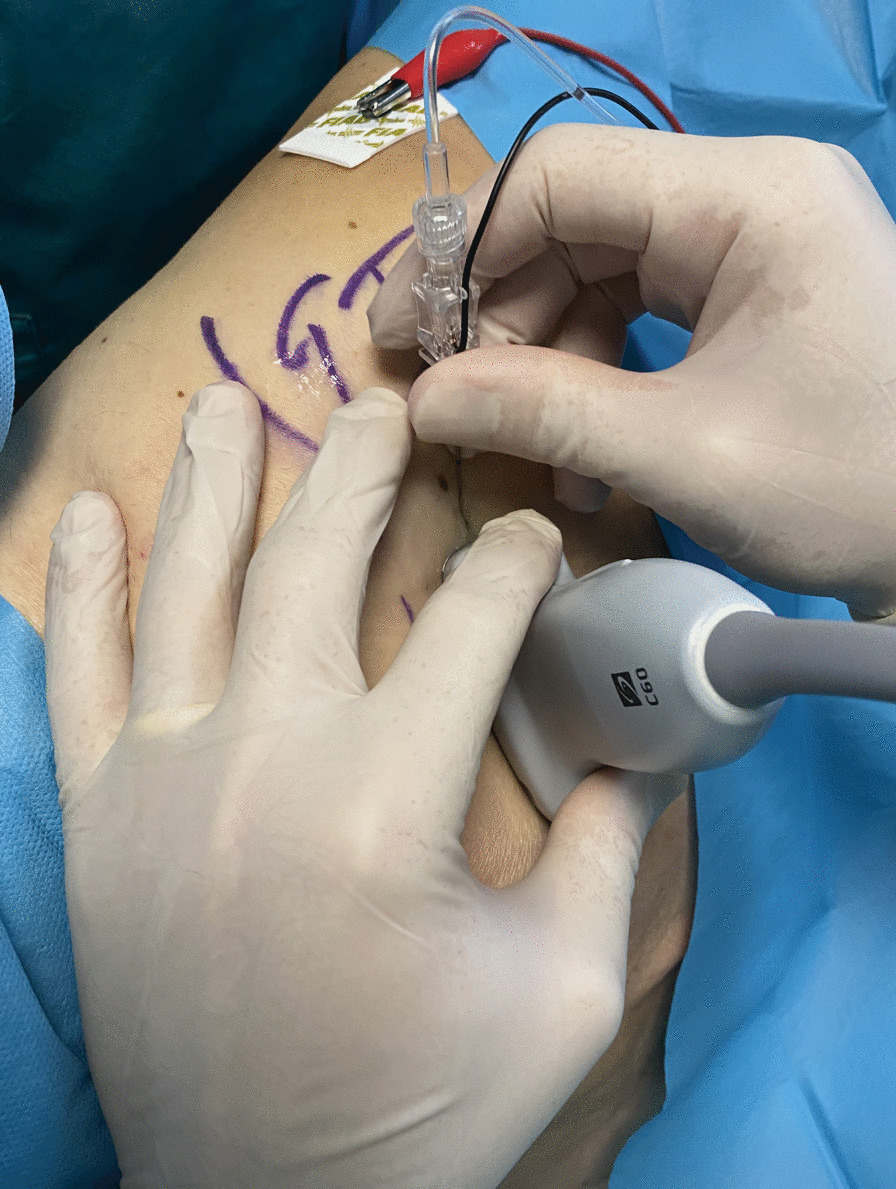
Ultrasound Sciatic Nerve block—Parasacral approach Greater trochanter (GT)

Lastly, we performed the Sciatic Nerve Block with a Parasacral approach to benefit from the analgesia provided by the blocks already performed. The patient was positioned in lateral decubitus, with the femur fracture limb positioned superiorly. The sacral region was disinfected and sterile towels were placed to delimit the field. A convex probe was used. A line, which connected the Greater Trochanter and the Postero-Superior Iliac Spine, was drawn. Along this line, with appropriate pressure, rotation, and tilting movements, the various structures could be identified, including the Piriformis Muscle, with a hypoechoic appearance, and the Sciatic Nerve, with a typical "honeycomb" appearance.

From the lateral side of the transducer (lateral-to-medial orientation), a 100-mm long 21-Gauge 30° tip (Vygon Value Life, Italy) was inserted to ensure complete visualization of the needle tip. The correct position of the needle tip was double-checked through the progressive injection of 3 ml of Saline Solution. Electrical nerve stimulation (ENS) assisted technique was performed to confirm the target nerve. A stimulating current was used (0.4 mA), when the needle was near the target nerve and the lowest twitch response was seen or paresthesia was felt. An anesthetic solution of Ropivacaine 0.375% (dose 37.5 mg) plus Mepivacaine 1% (dose 100 mg) and Dexamethasone 4 mg, total volume 21 ml, were later injected.

At the end of the execution of the ultrasound PNB, the motor and sensory blocks were tested, and evaluated respectively with the " Bromage scale" and with the " Hollmen scale". All the patients had an adequate anesthetic plane with a complete motor block (Bromage scale 1) and sensory block tested via pinprick and ice tests that showed sensation loss (Hollmen Scale 4). All patients were administered an interview at the end of surgery which showed they tolerated the anesthesiological procedure excellently without any pain or discomfort throughout the surgical phase.

### Post-operative management

The patients were evaluated by clinicians every 6 h in each postoperative period (first 24 h) to determine: VAS; the presence of adverse effects such as nausea, vomiting, pruritus, shivering, and motor recovery time. Pharmacological therapy was based on the patient's response. In the postoperative period, VAS assessment was carried out with a 10 cm long line with verbal anchors at either extremities (“no pain” on the far left and “the most intense pain” on the far right). The patient marked a point on the line corresponding to the rating of pain intensity. After surgery, we administered intravenous Paracetamol 1 g 3 times a day. Oxycodone (up to 0.1 mg/Kg) was available as a rescue dose. Pain control was considered good in the case of a VAS score of less than 4.

No patient required a rescue dose and no side effect was observed. In the 24 h following the operation, no patient required the use of rescue therapy. The median (range) times to full recovery from motor blockade was 12 (4–18) hours and 10 (4–12) hours for sensory blockade.

### Follow up

On the first day after surgery, rehabilitation was initiated and training for standing was possible. Eventually, they were transferred to a rehabilitation hospital on the 15th day after surgery.

These types of ultrasound-guided PNBs were appropriate for the procedure, showing fewer risks or side effects and this anesthesiologic management is replicable.

## Discussion

The presence of numerous comorbidities and contraindications addressed us to the choice of anesthesiologic conduct with PNB. As far as we know, this is the first study that shows this anesthesiologic approach for surgical FF treatment. We define “Tetra-Block” as the four performed ultrasound PNBs. As to the first patient, GA appeared to be contraindicated due to the comorbidities. She presented a pulmonary condition that required suitable evaluations, both in terms of airway management and for any post-operative complications [13]. COPD is associated with increased morbidity, mortality, and length of stay [14]. GA appears to be an independent risk factor in the development and worsening of dementia [15]. The choice of GA was precluded for the second patient due to his categorical refusal. The third patient had lung metastasis and several randomized controlled trials were in progress to provide a better understanding of how volatile and intravenous hypnotics impact influenced cancer progression and to evaluate the effect of the anesthesia techniques on the immune system and tumor microenvironment [16].

Spinal anesthesia (SA) was the first choice for FF. It reduced the risk of postoperative complications such as hypoxia, myocardial infarction, deep vein thrombosis, pneumonia, pulmonary embolism, and delirium [17]. SA, in this case, was not possible due to some absolute contraindications: as to the first patient, a nephropathic subject, NAO therapy was not suspended, therefore blood coagulation framework was compromised; as regards the second patient, it was not possible to proceed with SA due to severe Aortic Stenosis. In the case of Aortic Stenosis, it has been demonstrated that it is still possible to carry out a NA using a Low-Dose Spinal [18]. This was not possible in our management due to technical difficulties of approach and surgical times.

The possibility of operating with a loco-regional approach is supported by the literature, and the outcome of frail patients treated with PNB compared to those subjected to GA is better, both in terms of mortality and hospitalization time [19]. The literature testifies how hip and femur surgery can be performed with the execution of a Lumbar Plexus Block, associated or not with the Sciatic Nerve Block [20]. ESAIC/ESRA guidelines defined that deep nerve procedures, like Lumbar Plexus block, should be performed according to the recommendations for neuraxial procedures. If the INR is not below the minimum recommended level, regional anesthetic management should depend on the compressibility of the puncture site, the vicinity of (large) blood vessels, and/or neuraxial structures [21]. In such fragile patients, an opioid-free anesthetic technique can be used, considerably reducing the impact of adverse events and side effects of the use of opioids, but not eliminating adverse hemodynamic events [22]. You can adapt your anesthesiological management to every patient's needs.

Our anesthesiologic approach to ultrasound PNB, named “Tetra-block”, has some critical issues. First of all, multiple blocks are necessary to ensure an anesthesiologic plane for the procedure, with the need to have operators with considerable experience in the LRA. The execution by operators with little experience exposes us to the risk of LAST,^10^ especially in patients with severe hypoalbuminemia or debilitated, or of inadequate anesthesia, with poor satisfaction by the patient and the surgical team. It is to be noted that ropivacaine 75 mg and mepivacaine 200 mg were the total dose. This dose is very far from the toxic dose for ropivacaine (3 mg/kg) and mepivacaine (6 mg/kg). Like all ultrasound nerve blocks, they are an alternative anesthesiologic strategy in many situations but do not forget that they need competence, ability, experience, and appropriateness. Last but not least, the cooperation of the surgical team; even the position of the patient can be difficult, the other lower limb is not anesthetized and patients often suffer from hip arthrosis. On the other hand, the execution of these PNBs does not involve the suspension of anticoagulant and antiplatelet drugs. Considering the various anesthetic alternatives, it was decided to proceed with the loco-regional approach.

## Conclusions

Our report illustrates the use of PNB with minimally invasive hemodynamic monitoring as a valid alternative to spinal anesthesia in patients with FF who were undergone urgent surgery. The flexibility that can be achieved with LRA must prompt us to reflect on what the actual usage scenarios might be. It must be seen as an additional option in our anesthetic arsenal. This type of approach may be useful in patients where drug therapy cannot be optimized, as in antiplatelet and anticoagulant therapy. It is always necessary to carefully evaluate any limitations related to patient compliance, the risk of Systemic Intoxication from Local Anesthetics, and any hemodynamic repercussions.

## Study design

We presented a series of three clinical cases in the urgent setting, in which we chose a locoregional anesthesia plan with four different peripheral nerve blocks that we called "Tetra-block”.

## Data Availability

All data generated or analyzed during this study are included in this published article.

## Abbreviations

FF: Femur fracture
GA: General anesthesia
NA: Neuraxial anesthesia
LRA: Locoregional anesthesia
PNB: Peripheral nerve blocks
RA: Regional anesthesia
PACU: Post- anesthesia care unit
PONV: Postoperative nausea and vomiting
RA: Regional anesthesia
LAST: Local anesthetic systemic toxicity
BMI: Body mass index
COPD: Chronic obstructive pulmonary disease
EF: Ejection fraction
NAO: Novel oral anticoagulants
DAPT: Dual antiplatelet therapy
IDC: Invasive ductal carcinoma
SA: Spinal anesthesia
ENS: Electrical nerve stimulation

## References

[1] Cummings SR, Melton LJ (2002). Epidemiology and outcomes of osteoporotic fractures. Lancet.

[2] Grisso JA, Kelsey JL, Strom BL (1991). Risk factors for falls as a cause of hip fracture in women. The Northeast Hip Fracture Study Group. N Engl J Med..

[3] Kanis JA, Odén A, McCloskey EV, Johansson H, Wahl DA, Cooper C, IOF Working Group on Epidemiology and Quality of Life (2012). A systematic review of hip fracture incidence and probability of fracture worldwide. Osteoporos Int..

[4] Katsoulis M, Benetou V, Karapetyan T (2017). Excess mortality after hip fracture in elderly persons from Europe and the USA: the CHANCES project. J Intern Med.

[5] Dyer SM, Crotty M, Fairhall N (2016). Fragility Fracture Network (FFN) Rehabilitation Research Special Interest Group. A critical review of the long-term disability outcomes following hip fracture. BMC Geriatr..

[6] Italian Society of Orthopaedics and Traumatology. Proximal femur fractures in the elderly. SIOT guideline 2021. https://siot.it/lineeguida-fratturafemore2021

[7] Liu JL, Wang XL, Gong MW (2014). Comparative outcomes of peripheral nerve blocks versus general anesthesia for hip fractures in geriatric Chinese patients. Patient Prefer Adherence.

[8] Khan SA, Qianyi RL, Liu C, Ng EL, Fook-Chong S, Tan MG (2013). Effect of anaesthetic technique on mortality following major lower extremity amputation: a propensity score-matched observational study. Anaesthesia.

[9] Coviello A, Vargas M, Castellano G, Maresca A, Servillo G (2020). Ultrasound-guided Erector Spinae Plane Block (US-ESPB)-anesthetic block: case report. Clin Case Rep.

[10] Drasner K (2010). Local anesthetic systemic toxicity: a historical perspective. Reg Anesth Pain Med..

[11] Muller ME, Nazarian S, Koch P, Schatzker J (1990). The Comprehensive Classification of Fractures of Long Bones/AO Classification of Fractures.

[12] Meinberg EG, Agel J, Roberts CS, Karam MD, Kellam JF (2018). Fracture and dislocation classification compendium—2018. J Orthop Trauma..

[13] Coviello A, Golino L, Posillipo C, Marra A, Tognù A, Servillo G (2022). Anesthetic management in a patient with Arnold-Chiari malformation type 1,5: a case report. Clin Case Rep..

[14] Gupta H, Ramanan B, Gupta PK, Fang X, Polich A, Modrykamien A, Schuller D, Morrow LE (2013). Impact of COPD on postoperative outcomes: results from a national database. Chest.

[15] Sohn JH, Lee JJ, Lee SH (2021). Longitudinal study of the association between general anesthesia and increased risk of developing dementia. J Pers Med.

[16] Ramirez MF, Cata JP (2021). Anesthesia techniques and long-term oncological outcomes. Front Oncol.

[17] White SM, Altermatt F, Barry J (2018). International Fragility Fracture Network Delphi consensus statement on the principles of anaesthesia for patients with hip fracture. Anaesthesia.

[18] Coviello A, Ianniello M, Spasari E (2021). Low-dose spinal and opioid-free anesthesia in patient with Severe Aortic Stenosis and SARS-CoV-2 infection: case report. Clin Case Rep..

[19] Desai V, Chan PH, Prentice HA (2018). Is anesthesia technique associated with a higher risk of mortality or complications within 90 days of surgery for geriatric patients with hip fractures?. Clin Orthop Relat Res.

[20] Ahamed ZA, Sreejit MS (2019). Lumbar Plexus Block as an Effective Alternative to Subarachnoid Block for Intertrochanteric Hip Fracture Surgeries in the Elderly. Anesth Essays Res..

[21] Kietaibl S, Ferrandis R, Godier A, Llau J, Lobo C, Macfarlane AJ, Schlimp CJ, Vandermeulen E, Volk T, von Heymann C, Wolmarans M, Afshari A (2022). Regional anaesthesia in patients on antithrombotic drugs: Joint ESAIC/ESRA guidelines. Eur J Anaesthesiol.

[22] Coviello A, Golino L, Maresca A, Vargas M, Servillo G (2021). Erector spinae plane block in laparoscopic nephrectomy as a cause of involuntary hemodynamic instability: A case report. Clin Case Rep..

